# Improving Cofactor Promiscuity of HMG-CoA Reductase from *Ruegeria pomeroyi* Through Rational Design

**DOI:** 10.3390/biom15070976

**Published:** 2025-07-07

**Authors:** Haizhao Xue, Yanzhe Huang, Aabid Manzoor Shah, Xueying Wang, Yinghan Hu, Lingyun Zhang, Zongbao K. Zhao

**Affiliations:** 1 Dalian Institute of Chemical Physics, Chinese Academy of Sciences, Dalian 116023, China; xuehaizhao@dicp.ac.cn (H.X.); huangyz@dicp.ac.cn (Y.H.); aabidmanzoor87@gmail.com (A.M.S.); wangxueying@dicp.ac.cn (X.W.); huyinghan@dicp.ac.cn (Y.H.); zhanglingyun21@dicp.ac.cn (L.Z.); 2 University of Chinese Academy of Sciences, Beijing 100049, China; 3 School of Bioengineering, Dalian University of Technology, Dalian 116024, China

**Keywords:** mevalonate pathway, 3-hydroxy-3-methylglutaryl-coenzyme A reductase, rational design, dual-cofactor specificity

## Abstract

The mevalonate pathway is crucial for synthesizing isopentenyl pyrophosphate (IPP), the universal precursor of terpenoids, with 3-hydroxy-3-methylglutaryl-CoA reductase (HMGR) serving as the rate-determining enzyme that catalyzes the reduction of 3-hydroxy-3-methylglutaryl-CoA (HMG-CoA) to mevalonate, requiring NAD(P)H as an electron donor. Improving the cofactor promiscuity of HMGR can facilitate substrate utilization and terpenoid production by overcoming cofactor specificity limitations. In this study, we heterologously expressed *rp*HMGR from *Ruegeria pomeroyi* in *Escherichia coli* BL21(DE3) for the first time and established that it predominantly utilizes NADH. To broaden its cofactor usage, we employed Molecular Operating Environment (MOE)-assisted design to engineer the cofactor binding site, creating a dual-cofactor-utilizing mutant, D154K (the substitution of aspartic acid with lysine at residue 154). This mutant exhibited a significant 53.7-fold increase in activity toward NADPH, without compromising protein stability at physiological temperatures. The D154K mutant displayed an optimal pH of 6, maintaining over 80% of its catalytic activity across the pH range of 6–8, regardless of whether NADH or NADPH was the cofactor. These findings highlight the value of rational design, enhance our understanding of HMGR-cofactor recognition mechanisms, and provide a foundation for future efforts to optimize and engineer HMGR for broader cofactor flexibility.

## 1. Introduction

Isoprenoid compounds, also known as terpenoids, form a diverse class of natural products with a wide range of biological activities and physicochemical properties. To date, over 100,000 terpenoids have been identified, accounting for nearly one-third of the entries in the Dictionary of Natural Products (accessed on 1 January 2025; http://dnp.chemnetbase.com) [1,2,3]. Terpenoids exhibit diverse functionalities and are extensively utilized in pharmaceuticals, fragrances and flavorings, and biofuels, demonstrating significant industrial importance [4,5,6,7]. Research on the production of terpenoids using microbial cell factories has become a prominent topic in synthetic biology in recent years [8,9,10]. Isopentenyl pyrophosphate (IPP) is the precursor for the biosynthesis of all terpenoid compounds, with its production primarily occurring through two pathways, the mevalonate pathway (MVA) and the methylerythritol phosphate pathway (MEP). The MVA pathway is found in all eukaryotes, as well as in certain archaea and bacteria [11,12]. 3-Hydroxy-3-methylglutaryl-coenzyme A reductase (HMGR) catalyzes the sole redox reaction in the mevalonate pathway and acts as its rate-limiting enzyme [13]. Beyond its role as a key regulatory protein in the terpenoid biosynthesis pathway, HMGR also serves as a target for drugs such as statins [14,15], rendering research on HMGR of significant importance.

**Figure 1:**
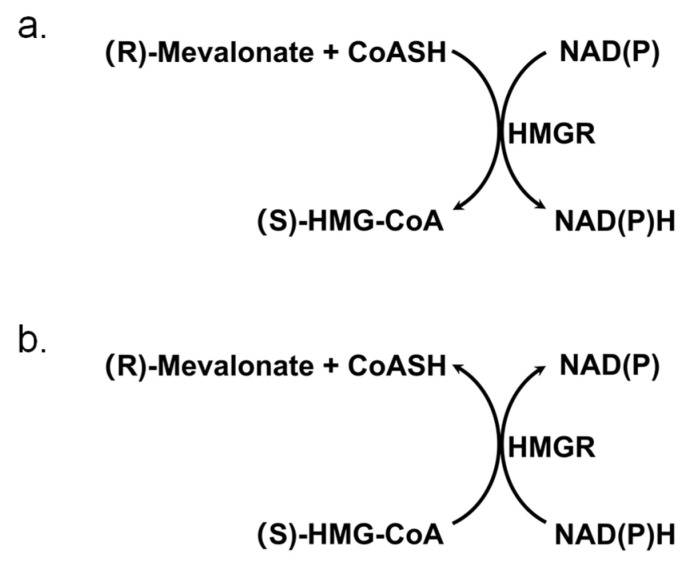
Schematic diagram of the catalytic reaction by *rp*HMGR. (**a**) Schematic diagram of the reverse reaction (oxidative acylation of mevalonate); (**b**) schematic diagram of the forward reaction (reductive deacylation of HMG-CoA).

Similar to many oxidoreductases, the reaction catalyzed by HMGR requires cofactors such as NAD(H) or NADP(H) as electron transfer mediators. However, unlike the reactions catalyzed by most oxidoreductases, HMGR catalyzes a complex four-electron redox process [16], with its reaction equation as follows and also in Figure 1:


(S)-HMG-CoA + 2NAD(P)H + 2H ⇌ (R)-mevalonate + 2NAD(P) + CoASH

Based on sequence and evolutionary analyses of HMGR, it has been classified into two major categories [17,18]. Class I HMGR is membrane-bound, featuring both transmembrane and catalytic domains. Genes encoding Class I HMGR are predominantly found in eukaryotes and primarily depend on NADPH. In contrast, Class II enzymes lack transmembrane domains [19]. Genes encoding Class II HMGR are present in bacteria and some archaea, some of which are strictly dependent on NADH (e.g., HMGR from *Pseudomonas mevalonii* and *Burkholderia cenocepacia*) [20,21,22], while others mainly depend on NADPH (e.g., HMGR from *Enterococcus faecalis*, *Staphylococcus aureus*, *Listeria monocytogenes*, and *Streptococcus pneumoniae*) [23,24,25,26,27,28]. The crystal structures of Class I HMGR from humans and Class II HMGR from *P. mevalonii*, which was the first Class II enzyme identified [29,30], were among the first to be resolved [31,32]. These structures reveal that the minimal functional unit of HMGR is a dimer, with its catalytic active site located at the dimer interface. Electron transfer occurs with the assistance of the nicotinamide cofactor NAD(P)H, and both classes of HMGR share similar catalytic mechanisms. Each monomer of Class II HMGR consists of the following three structural domains: a large domain that binds HMG-CoA, a small domain that binds NAD(H), and a C-terminal flap domain. Conserved residues Glu, Asp, Lys, and His are directly involved in or assist with electron transfer during the redox reaction [16,18,31].

The reaction catalyzed by HMGR relies on NAD(P)H as the electron transfer mediator. Consequently, its catalytic efficiency is constrained by the level and state of NAD(P)H in the cell. When constructing microbial cell factories for terpenoid synthesis, HMGR is commonly overexpressed to strengthen the mevalonate pathway [33], thereby ensuring sufficient amounts of the precursor substance IPP. However, this may lead to excessive consumption of specific cofactors, which is detrimental to maintaining metabolic homeostasis and achieving high terpenoid yields. Enhancing the cofactor promiscuity of HMGR can make its cofactor selection more flexible under different physiological states and metabolic environments. This can reduce the adverse effects of fluctuations in specific cofactors on the mevalonate pathway, thereby improving substrate utilization efficiency and terpenoid production. Although some reported HMGR enzymes can utilize both NADH and NADPH as cofactors, there are substantial differences in the utilization abilities of the same enzyme for NADH and NADPH. For example, HMGR from *Archaeoglobus fulgidus* has a 6.3-fold higher utilization capacity (*k*cat/*K*m) for NADH compared with NADPH [27], whereas HMGR from *S. aureus* exhibits approximately a 35-fold higher utilization of NADPH compared with NADH [25]. Currently, no HMGR has been found to possess high activity toward both NADH and NADPH simultaneously.

The aim of this study was to obtain HMGR with dual-cofactor utilization capability. To achieve this, we first expressed recombinant HMGR from *Ruegeria pomeroyi*, a widely used biocatalyst, in *Escherichia coli* BL21(DE3); optimized the expression conditions; and successfully obtained a stable protein. Using multiple sequence alignment, MOE-aided design, and structural simulations, we then rationally modified the cofactor binding site of *rp*HMGR, resulting in the D154K mutant, which exhibits dual-cofactor utilization. The properties of both *rp*HMGR and the D154K mutant were further investigated. Through this recombinant expression and rational modifications, our understanding of the recognition mechanism between HMGR and its cofactors has been deepened.

## 2. Materials and Methods

### 2.1. Strains, Plasmids, and DNA

The strains and plasmids used in this study are listed in Supplementary Table S1. *E. coli* BL21(DE3) was employed for the expression of recombinant proteins. The *E. coli* strains were first cultured in an LB medium (10 g/L tryptone, 5 g/L yeast extract, and 10 g/L NaCl) at 37 °C for activation. For protein expression, cultures were transferred to a TB medium (12 g/L tryptone, 24 g/L yeast extract, 5 g/L glycerol, 2.2 g/L KH_2_PO_4_, and 9.4 g/L K_2_HPO_4_) and incubated at either 30 °C or 18 °C. The medium was supplemented with 0.1 mmol/L IPTG to induce protein expression, and 50 μg/mL kanamycin was added to maintain selective pressure for plasmid retention.

The DNA sequence encoding *rp*HMGR was synthesized by General Biosystems (Chuzhou, China) and cloned into the pET28a(+) expression vector. The primers used for cloning the gene into the vector are detailed in Supplementary Table S2. The full sequence of *rp*HMGR is provided in Supplementary Table S3.

### 2.2. Reagents and Kits

Molecular cloning kits were purchased from Sangon Biotech (Shanghai, China). Isopropyl-β-D-thiogalactoside (IPTG), kanamycin, NAD, NADP, NADH, NADPH, Tris, HEPES, tryptone, agarose, lysozyme, DNase I, SDS, acrylamide, N, N′-methylenebisacrylamide, and Coomassie Brilliant Blue R-250 were obtained from DingguoChangsheng (Beijing, China). PrimeSTAR Max DNA polymerase, PrimeSTAR HS DNA polymerase, and *Dpn*I were sourced from Takara (Dalian, China). Protein marker, His-Tag mouse monoclonal antibody, and horseradish-peroxidase-labeled goat anti-mouse IgG (H+L) were purchased from Beyotime Biotechnology (Shanghai, China). Goldview nucleic acid dye and a DNA marker were acquired from TransGen (Beijing, China). The ECL chemiluminescence reagent was obtained from Tanon (Shanghai, China). (R, S)-HMG-CoA and (R, S)-mevalonate were purchased from Sigma-Aldrich, and coenzyme A was sourced from Yuanye (Shanghai, China).

### 2.3. Vector Construction and Mutant Construction

The *rp*HMGR-encoding gene was synthesized based on the codon preference of *E. coli* and subsequently cloned into pET28a(+), resulting in pET28a-*rp*HMGR(C). The vector pET28a-*rp*HMGR(N) was constructed with the Restriction-Free cloning method [34,35] using pET28a-*rp*HMGR(C) as the template. The primers (-pET) *rp*HMGR-N-his-F1 and (-pET) *rp*HMGR-N-HISR were used in the cloning process. The vector was electrotransformed into BL21(DE3) cells and verified by PCR and gene sequencing.

Using the plasmid pET28a-*rp*HMGR(N) as the template, D154K mutation was introduced via the Restriction-Free cloning method. The PCR (RF2) products were treated with a *Dpn*I enzyme to digest the parental template DNA. The digested products were then electrotransformed into BL21(DE3). The mutant was electrotransformed into BL21(DE3) cells and verified using gene sequencing by Sangon Biotech.

### 2.4. Construction of Mutant Libraries

The Restriction-Free cloning method was used for the library and plasmid constructions [36]. To construct the libraries, degenerated primers (Table S2) were designed by replacing the target codon with the degenerate codon NNK (N = A, T, G, or C and K = G or T, encoding the usual 20 amino acids). The corresponding primers and template plasmid were used to obtain fragments containing mutagenic modifications by PCR. The resulting fragments were used as mega primers to generate a nicked plasmid containing the corresponding mutations. The PCR products after RF2 were treated with *Dpn*I at 37 °C and transformed into electrocompetent *E. coli* BL21(DE3) cells. The quality of the library was evaluated by DNA sequencing. With 95% theoretical library coverage, 94 colonies were cultivated and the crude enzyme solution was verified.

### 2.5. Preparation of Crude Protein Enzyme Extract

BL21(DE3) samples containing the wild-type *rp*HMGR expression vectors pET28a-*rp*HMGR(C) and pET28a-*rp*HMGR(N) were induced in a TB medium at 30 °C and 200 rpm for 48 h, respectively. Three parallel cultures were prepared for each strain. BL21(DE3) containing the empty vector pET28a(+) was set as the negative control. The cells were lysed by a cell lysis buffer (10 mmol/L Tris-HCl, 1 mmol/L MgCl_2_, 1 mg/mL lysozyme, and 0.1 mg/mL DNase I; pH8.0) and the supernatant was collected for SDS-PAGE, Western blot, and crude enzyme activity assays.

### 2.6. SDS-PAGE and Western Blot

The crude enzyme solution samples were subjected to electrophoresis in a 12% SDS-polyacrylamide gel and then transferred onto a nitrocellulose membrane (PALL). After washing, the membrane was blocked with BSA at 37 °C for 1 h, followed by incubation with a His-Tag mouse monoclonal antibody (Beyotime, Shanghai, China) and, further, with a horseradish peroxidase (HRP)-labeled goat anti-mouse IgG (H+L) secondary antibody (Beyotime). Finally, the membrane was developed using an ECL chemiluminescent substrate (Tanon, Shanghai, China), and the results were captured by an automated chemiluminescence imaging system (Tanon-5200 Multi, Tanon, Shanghai, China).

### 2.7. Protein Expression and Purification

For *rp*HMGR expression, *E. coli* BL21(DE3) cells harboring expression vectors were cultured in an LB medium supplemented with 50 μg/mL kanamycin at 37 °C for 16 h. Then, it was inoculated at a 1:50 dilution into a TB medium containing 50 μg/mL kanamycin and cultured at 37 °C until its OD_600_ reached approximately 1. Afterwards, the solution was supplemented with 0.1 mmol/L IPTG and induced at 18 °C for 12 h. The cells were harvested by centrifugation (8000× *g*; 5 min), washed with ddH_2_O, and resuspended in a lysis buffer (50 mmol/L Tris-HCl, 200 mmol/L NaCl, 10% glycerol, 0.5 mmol/L PMSF, and 10 μg/mL DNase I; pH8.0).

For *rp*HMGR purification, the cells were lysed by sonication in an ice bath and the supernatant was collected by centrifugation (24,000× *g;* 30 min). Then, it was applied to a Ni-NTA chromatography column. The column was washed with an NWB washing buffer (50 mmol/L NaH_2_PO_4_, 0.5 mol/L NaCl, and 40 mmol/L imidazole; pH8.0) to remove impurities and then eluted with an NEB elution buffer (50 mmol/L NaH_2_PO_4_, 0.5 mol/L NaCl, and 250 mmol/L imidazole; pH8.0) to collect the protein solution, which was then concentrated by ultrafiltration. The molecular weight and purity of the purified protein were determined by SDS-PAGE.

### 2.8. Protein Rational Design

Sequence alignment was carried out by SnapGene (Dotmatics, Boston, MA, USA) using the MAFFT model.

Homology modeling, molecular docking, virtual screening, structural simulations, and ligand interaction analyses were performed using MOE (version 2022.02) (Chemical Computing Group ULC, Montreal, QC, Canada) software.

A homology model of *rp*HMGR was constructed using *pm*HMGR from P. mevalonii (PDB: 4I4B) as the template. Molecular docking was conducted to predict the binding mode of NADP to the modeled *rp*HMGR structure, in which the docking site was restricted at the cofactor binding pocket while NADP was used as the ligand. Triangle Matcher and Rigid Receptor were applied as the placement and refinement methods, respectively, during the docking process. After that, London dG with 100 poses and GBVI/WSA dG with 30 poses were used as score methods. Virtual screening focused on residue 154 (D154) of *rp*HMGR, where amino acid substitutions were scanned using MOE’s Residue Scan function. The scanning range included all 20 canonical amino acids, and the LowMode method was applied for conformational sampling. The dAffinity and dStability values, two important parts of the scoring system, of all mutants toward NADP were evaluated. Structural simulations and ligand interactions between the protein and cofactor of both the wild-type and the mutant were analyzed. Subsequently, the Protein Builder tool in MOE was utilized to modify selected amino acid residues, after which the most stable conformations were selected and subjected to energy minimization.

### 2.9. Activity Assay

For the crude enzyme activity, the reverse reaction (oxidative acylation of mevalonate; Figure 1a) was detected at 30 °C using the following reaction system: 50 mmol/L Tris-HCl, 1 mmol/L NAD, 2 mmol/L (R, S)-mevalonate, and 1 mmol/L coenzyme A; pH8.0. The absorbance change was continuously monitored at 340 nm. Each group included three parallel replicates.

For the specific activity determination of the purified protein, the forward reaction (reductive deacylation of HMG-CoA) and the reverse reaction (oxidative acylation of mevalonate) were detected at 30 °C. For the forward reaction, the assay buffer contained 50 mmol/L Tris-HCl, 50 mmol/L NaCl, 5 mmol/L DTT, 0.5 mmol/L (R, S)-HMG-CoA, and 0.5 mmol/L NADH or NADPH; pH7.5. For the forward reaction, the assay buffer contained 50 mmol/L Tris-HCl, 1 mmol/L NAD, 2 mmol/L (R, S)-mevalonate, and 1 mmol/L coenzyme A; pH8.0. The absorbance change was continuously monitored at 340 nm. Each group included three parallel replicates. One unit (U) of enzyme activity was defined as the amount of enzyme required to consume 1 μmol of NAD(P)H or NAD(P) per minute under the specified conditions.

### 2.10. Thermal Stability Analysis

The purified *rp*HMGR-WT and D154K enzymes were diluted to 0.5 mg/mL using a buffer (50 mmol/L Tris-HCl; pH7.5). For stability assessments at varying temperatures, both the WT and D154K were incubated at 30 °C, 40 °C, 50 °C, and 60 °C for 1 h. For stability assessments at 37 °C, they were incubated at 37 °C for 2 to 12 h. The corresponding enzyme activities were monitored via the reverse reaction (Figure 1a) using the following assay system: 50 mmol/L Tris-HCl, 1 mmol/L NAD, 2 mmol/L (R, S)-mevalonate, and 1 mmol/L coenzyme A, pH8.0. The absorbance change was continuously monitored at 340 nm at 30 °C. The relative enzyme activities under different incubation conditions were normalized according to the activity of the non-incubated enzymes. Each sample was tested in triplicate, and the error was represented by a standard deviation.

### 2.11. pH Profile of rpHMGR

To assess the pH profile of rpHMGR, buffers with different pH values were used (pH4.0–5.0: acetate sodium acetate buffer; pH6.0–7.5: potassium phosphate buffer; pH8.0–8.5: Tris-HCl buffer; pH9.0–11.0: glycine-NaOH buffer). For the reverse reaction, the reaction system consisted of 100 mmol/L buffer, 1 mmol/L NAD or NADP, 2 mmol/L (R, S)-mevalonate, and 1 mmol/L coenzyme A; pH8.0. For the forward reaction, the reaction system consisted of 100 mmol/L buffer, 50 mmol/L NaCl, 5 mmol/L DTT, 0.5 mmol/L (R, S)-HMG-CoA, and 0.5 mmol/L NADH or NADPH; pH7.5. Enzyme activity was measured at 30 °C, and relative enzyme activity was normalized according to the highest activity observed. Each sample was tested in triplicate, and the error was represented by a standard deviation.

## 3. Results and Discussion

### 3.1. Expression of rpHMGR

**Figure 2:**
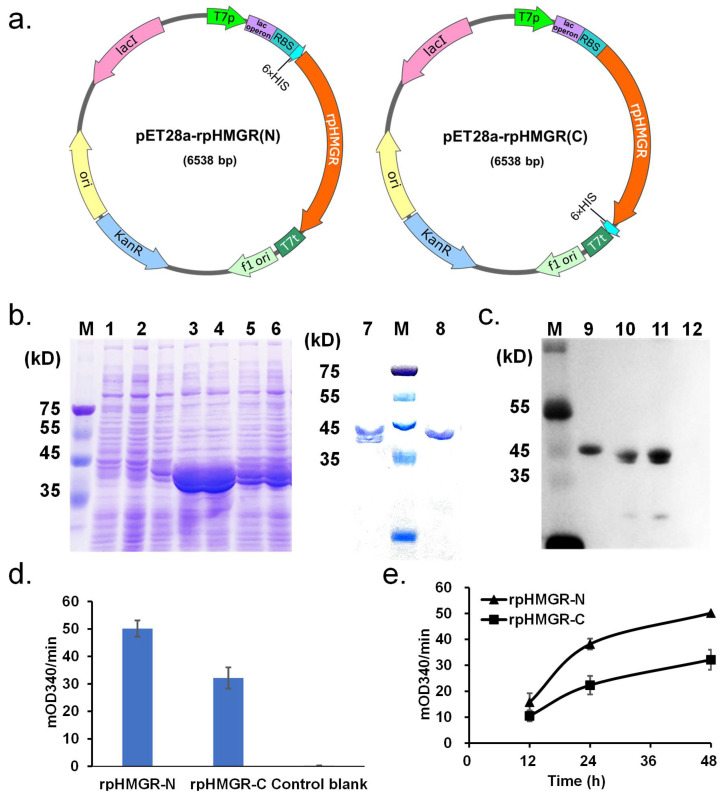
Expression and optimization of *rp*HMGR (**a**). Schematic diagram of expression vectors pET28a-*rp*HMGR(N) and pET28a-*rp*HMGR(C) with His-Tag fused to the N-terminus and C-terminus of *rp*HMGR protein, respectively. (**b**) SDS-PAGE verification of *rp*HMGR protein. Lanes 1 and 2: negative control with strain carrying empty pET28a(+) vector; lanes 3 and 4: crude enzyme solutions of *rp*HMGR-N; lanes 5 and 6: crude enzyme solutions of *rp*HMGR-C; lane 7: purified *rp*HMGR-N enzyme before optimization; lane 8: purified rpHMGR-N enzyme after optimization. (**c**) Western blot verification of *rp*HMGR crude enzyme solution. Lane 9: *rp*HMGR-C; lanes 10 and 11: *rp*HMGR-N; lane 12: negative control with strain carrying empty pET28a(+) vector. (**d**) Enzyme activity assay in crude enzyme solutions of different expression strains after 48 h of induction. “Control blank” refers to the negative control with the strain carrying the empty pET28a(+) vector. (**e**) Changes in enzyme activity in crude enzyme solutions of different expression strains over induction time. Experiments were performed in triplicate, and error bars indicate the standard deviation. The theoretical molecular size of *rp*HMGR is 45.42 kD. (original Western blot image see Supplementary Figure S1).

The coding DNA of *rp*HMGR (NCBI Accession Number: WP_011241944.1) was optimized and synthesized according to *E. coli* codon usage preferences. This optimized gene was then cloned into the pET28a(+) vector. A 6× His-Tag was fused either to the N-terminus (*rp*HMGR-N) or the C-terminus (*rp*HMGR-C) of the protein, resulting in the following two expression vectors: pET28a-*rp*HMGR(N) and pET28a-*rp*HMGR(C) (Figure 2a).

The expression of *rp*HMGR in *E. coli* strains carrying pET28a-*rp*HMGR(N) and pET28a-*rp*HMGR(C) vectors was assessed under induction conditions of 0.1 mmol/L IPTG at 30 °C for 48 h. Protein bands were observed in both *rp*HMGR-N and *rp*HMGR-C crude extracts, indicating expression was successful wherever the His-Tag was, while the expression level of *rp*HMGR-N was notably higher (Figure 2b). Further enzymatic activity assays showed the same trend during the 48 h induction period (Figure 2d,e).

SDS-PAGE analyses of both *rp*HMGR-N and *rp*HMGR-C crude enzyme extracts revealed two bands near 45 kD, corresponding with the target protein, in addition to a lower molecular weight band. This suggested partial protein degradation or incomplete expression, potentially due to issues with start codon recognition. To further investigate, a Western blot analysis was performed on both *rp*HMGR-N and *rp*HMGR-C crude extracts to identify which protein segments were affected. The results showed that both bands in the *rp*HMGR-N extract were able to bind to the His antibody, whereas in the *rp*HMGR-C extract, only the higher molecular weight band bound to the His antibody (Figure 2c, uncropped blot in Figure S1). This suggested that the lower molecular weight band in *rp*HMGR-C corresponded with a truncated protein, possibly due to incomplete expression at the C-terminus. Based on the known crystal structures of HMGR from other sources, it is hypothesized that the C-terminal flap domain in class II HMGR is a variable region, which often remains unobserved during protein crystallization. Notably, the flap domain is essential for HMGR catalytic activity, likely regulating substrate binding and release through conformational transitions and dynamic movements [24]. Therefore, the two bands observed were likely a result of degradation at the C-terminal flap domain.

To minimize protein degradation, we optimized the expression conditions by lowering the temperature and shortening the induction time. Specifically, the culture temperature was lowered from 30 °C to 18 °C and the induction time was reduced to 12 h. Under these optimized conditions (0.1 mmol/L IPTG at 18 °C for 12 h), purified *rp*HMGR-N retained only one single band at the correct molecular weight position, indicating the successful purification of the desired protein (Figure 2b, lane 8). In contrast, proteins induced under the original conditions (0.1 mmol/L IPTG at 30 °C for 48 h) showed signs of degradation (Figure 2b, lane 7). These results demonstrated that lowering the induction temperature and shortening the induction time were effective strategies for minimizing *rp*HMGR degradation.

### 3.2. Rational Design of Site-Directed Mutagenesis

The structure of *rp*HMGR has not yet been resolved; however, through sequence alignment, it was found that *pm*HMGR from *P. mevalonii* exhibited the highest sequence identity (61%) with *rp*HMGR among the available crystal structures. *R. pomeroyi* occupies an important ecological niche in marine systems, serving as a key microorganism for dimethylsulphoniopropionate (DMSP) degradation. Its functional role parallels that of *P. mevalonii* in ecosystems, with both genera participating in the breakdown of polycyclic aromatic hydrocarbons (PAHs) and other complex organics. This functional convergence likely drives the high homology between the two HMGRs. Therefore, *pm*HMGR (PDB ID: 4I4B) [37] was used as a template for the homology modeling of *rp*HMGR. The RMSD of the homology model relative to 4I4B was 0.06 Å. The resulting Ramachandran plot for the modeled *rp*HMGR structure indicated that all dihedral angles of amino acid residues fell within allowed regions, validating the high quality of the model (Figure S2).

**Figure 3:**
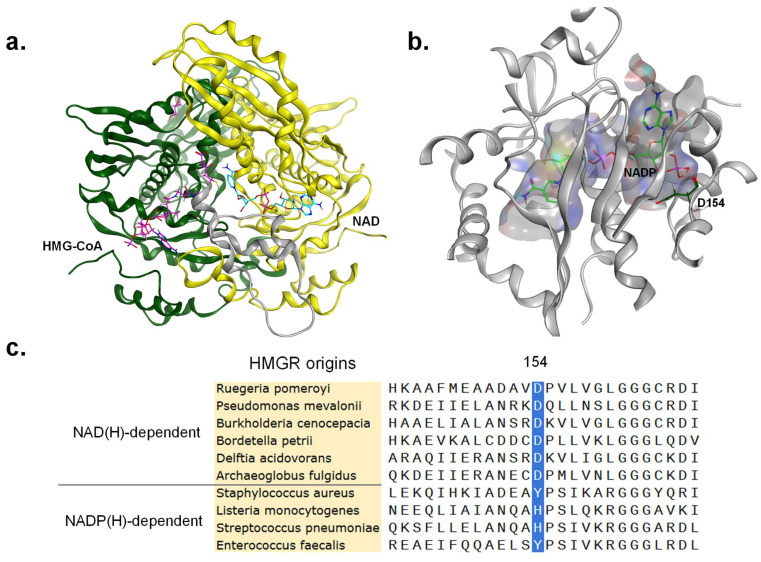
Homology modeling, molecular docking, and multiple sequence alignments: (**a**) structure of the homology-modeled *rp*HMGR (dimer) in a ternary complex with substrate HMG-CoA and cofactor NAD, where the magenta ligand represents HMG-CoA, and the cyan ligand denotes NAD; (**b**) molecular docking of *rp*HMGR with NADP, showing the cofactor binding region and the structure of NADP; (**c**) partial sequence alignment of NAD(H)/NADP(H)-dependent HMGRs from various species, with corresponding NCBI accession numbers (*Pseudomonas mevalonii* (PDB: 4I4B), *Burkholderia cenocepacia* (PDB: 6P7K), *Bordetella petrii* (WP_012250240.1), *Delftia acidovorans* (PDB: 6EEU), *Archaeoglobus fulgidus* (WP_010879232.1), *Staphylococcus aureus* (WP_029549965.1), *Listeria monocytogenes* (WP_003721392.1), *Streptococcus pneumoniae* (PDB: 5WPJ), *Enterococcus faecalis* (PDB: 7M1Z)).

Based on the homology modeling results, key conserved amino acids and structural domains were identified. The conserved amino acids were defined as E91, K275, D291, and H389 in *rp*HMGR, corresponding with E83, K267, D283, and H381 in *pm*HMGR, respectively. These residues, along with the functional domains, were mapped onto the primary structure. Residues 1–117 and 223–385 corresponded with the large domain binding to HMG-CoA, residues 118–222 with the small domain binding to NAD(H), and residues 385–433 with the C-terminal flap domain (Figure S2d). A homology-modeled structure of *rp*HMGR (dimer) in a complex with its substrate HMG-CoA and cofactor NAD is presented in Figure 3a. In this model, the substrate HMG-CoA and cofactor NAD are bound to the active site located at the interface between two monomers, which formed a homodimer, the smallest functional unit of HMGR. A further structural analysis was conducted by docking NADP with the *rp*HMGR model to examine the structural differences between the wild-type *rp*HMGR and NAD(P) (Figure 3b). Upon docking, it was observed that when the cofactor shifted from NAD to NADP, the hydrogen bond between D154 near the phosphate group of NADP and the adenosine ribose was lost. This change weakened the interactions between the protein and the cofactor. A multiple sequence alignment of NAD(H)- and NADP(H)-dependent HMGRs from various sources revealed that the NAD(H)-dependent enzymes exhibited conservation at residue position 154, which was consistently aspartate in all five aligned HMGRs. Although HMGR from *A. fulgidus* exhibits cofactor promiscuity toward both NAD(H) and NADP(H), it demonstrates a strong preference for NAD(H). This catalytic site similarly harbors an aspartate residue. In contrast, NADP(H)-dependent HMGRs displayed variability at this position: the four selected Class II HMGRs featured either tyrosine or histidine at this site (Figure 3c). Empirical insights from studies engineering cofactor preference in oxidoreductases further suggest that mutating the aspartate residue near the 2′-OH of NAD to a positively charged amino acid enhances NADP utilization capacity [38]. For the aforementioned reasons, it is hypothesized that the acid-base property of the amino acid at position 154 plays a crucial role in determining the cofactor preference of HMGR.

**Figure 4:**
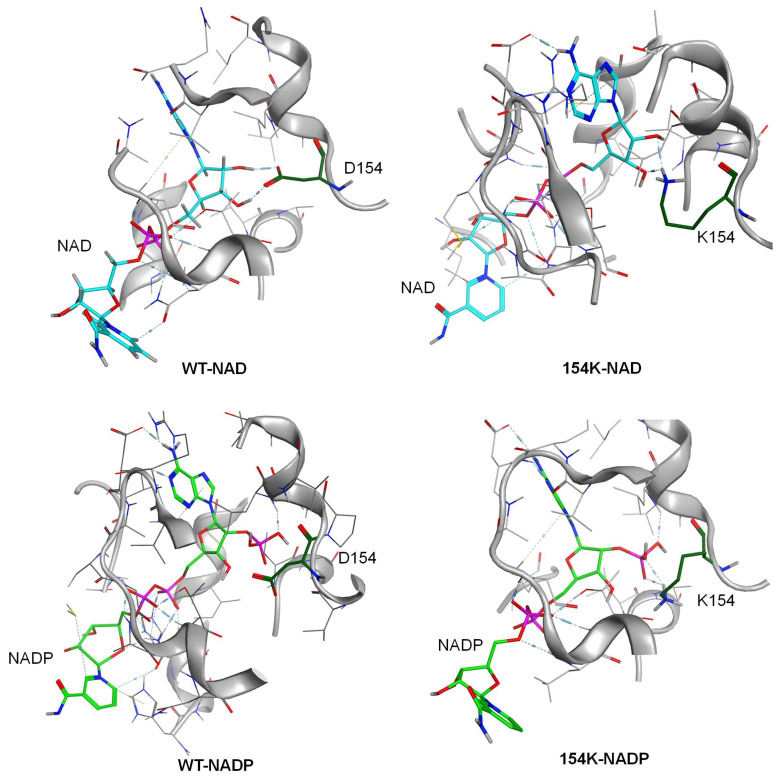
Structural analysis of cofactor binding domain of *rp*HMGR WT and D154K; structural analysis was based on homologous modeling and molecular docking by MOE. The cyan ligand represents NAD, the light green ligand denotes NADP, and the protein is shown in light gray with residue 154 highlighted in dark green.

Aware of the decisive role of residue 154 in cofactor recognition, virtual screening was then performed with MOE, as shown in Table S3. The results indicated that several mutants—including D154R and D154K substitutions with positively charged amino acids at position 154—exhibited enhanced affinity for NADP. Consequently, we simulated and analyzed mutant structures, revealing that lysine substitution enabled sterically favorable interactions with both NAD and NADP, and the mutation also strengthened the hydrogen bond network and optimized local electrostatic interactions. Following the docking of NADP into the cofactor binding pocket, aspartate at position 154 of the wild-type *rp*HMGR showed no interaction with the 2′-phosphate group of NADP; however, the D154K mutant formed additional hydrogen bonds with the 2′-phosphate. Notably, when NAD was bound as a cofactor, the lysine residue similarly established hydrogen bonds with both the 2′-OH and 3′-OH groups (Figure 4). These findings suggest that D154K may exhibit stronger affinity toward both NAD(H) and NADP(H), indicating its potential catalytic competence at utilizing both cofactors. Additionally, larger and bulkier arginine could create a greater impact on the protein architecture, the D154K mutant was prioritized as the initial candidate for experimental validation.

### 3.3. Activity Analysis of rpHMGR

**Figure 5:**
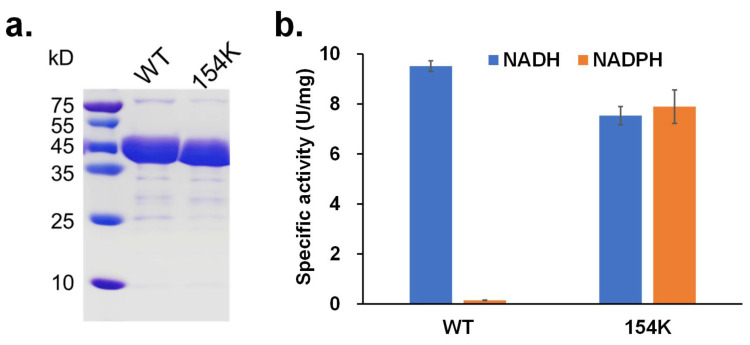
Enzyme activity and stability of wild-type and mutant *rp*HMGR. (**a**) SDS-PAGE of wild-type (WT) and D154K; (**b**) specific activities of wild-type (WT) and D154K toward NADH and NADPH. Experiments were performed in triplicate, and error bars indicate the standard deviation.

To verify our hypothesis, a D154K mutant was generated, expressed, and purified (Figure 5a). The enzymatic activity assays of both the wild-type *rp*HMGR (WT) and D154K mutant were performed with NADH and NADPH as cofactors, respectively. It was found that the wild-type *rp*HMGR primarily utilized NADH, with a specific activity of 9.51 U/mg under the tested conditions. In contrast, the activity with NADPH was minimal. However, the D154K mutant exhibited a significantly improved ability to utilize NADPH, achieving a specific activity of 7.89 U/mg, 53.7-fold higher than that of the wild-type enzyme (Figure 5b). Additionally, the D154K mutant retained the ability to utilize NADH, with a specific activity of 7.54 U/mg, which was comparable with the activity observed for NADPH. We further evaluated the activities in the reverse reaction (oxidative acylation of mevalonate) toward NAD and NADP. As shown in Figure S3, consistent with the reductive deacylation of HMG-CoA, the wild-type *rp*HMGR exhibited minimal activity toward NADP, whereas the D154K mutant demonstrated comparable activity toward both NAD and NADP. These findings confirm that residue 154 is a critical site for cofactor specificity, and the mutation of aspartic acid to lysine enhances cofactor promiscuity. The D154K mutant thus gains the ability to utilize both NADH and NADPH, demonstrating a broader cofactor utilization profile compared with the wild-type enzyme.

Furthermore, to identify superior variants at position 154, we performed saturation mutagenesis at this site. The constructed library achieved 95% coverage for 20 amino acids, and we subsequently analyzed the cofactor utilization capacity of crude enzyme extracts toward NAD and NADP within this library. However, no mutants demonstrating improved NADP activity or enhanced cofactor promiscuity compared with D154K were identified. Consequently, we focused the subsequent investigations on characterizing the D154K mutant.

### 3.4. Analysis of Structure–Activity Relationship in Mutants

**Figure 6:**
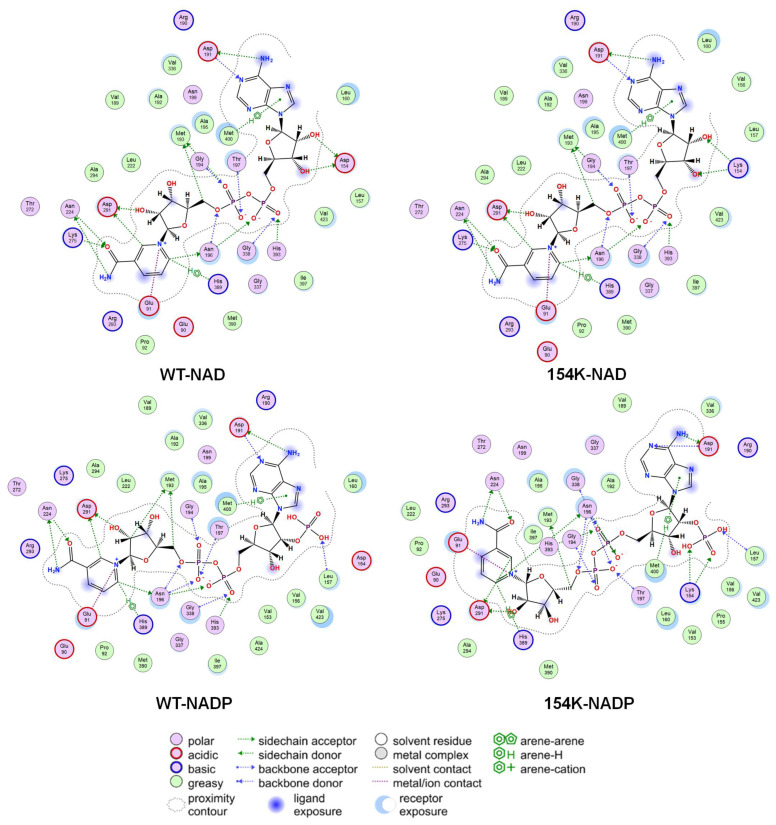
Interaction analysis between cofactor binding domain of *rp*HMGR wild-type and D154K with NAD(P). Interaction analysis was based on homologous modeling and molecular docking by MOE.

The mechanism underlying the changes in activity and cofactor preference in the D154K mutant was further investigated through structural modeling and interaction analyses. Although the introduction of D154K disrupted the hydrogen bonds between aspartic acid and the 2′- and 3′-hydroxyl groups of the adenine ribose in NAD, it allowed the re-establishment of a hydrogen bond between the side chain of lysine and the 2′- and 3′-hydroxyl group of the ribose, thus stabilizing the binding of NAD and allowing D154K to maintain NAD activity. Simultaneously, the introduction of lysine residues induced minimal perturbation on *rp*HMGR interactions within the NAD adenine and nicotinamide ribose. Moreover, when bound to NADP, D154K formed hydrogen bonds with the 2′-phosphate of NADP. The steric hindrance of the phosphate group oriented the lysine side chain’s amino group toward the ribose ring, which further strengthened the hydrogen bond network. This enhanced interaction between the lysine side chain and the ribose resulted in more stable binding between the protein and NADP. The K154 mutation preserved the ion contact between the catalytic residue E91 and the nicotinamide redox center of NAD(P) (Figure 6).

### 3.5. Analysis of Thermal Stability of Mutants

**Figure 7:**
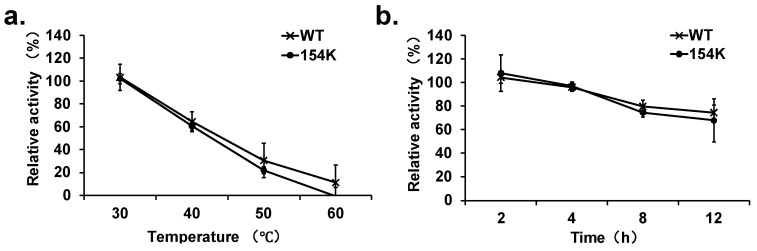
Thermal stability of wild-type (WT) *rp*HMGR and mutant D154K: (**a**) residual relative enzyme activity of protein samples after being treated at 30~60 °C for 1 h; (**b**) residual relative enzyme activity of protein samples after being treated at 37 °C for 2~12 h. Experiments were performed in triplicate, and error bars indicate the standard deviation.

To investigate the potential impact of the D154K mutation on protein stability, the thermal stability of D154K and the wild-type *rp*HMGR was assessed. MOE software predicted that the mutation at position 154 could influence the stability of the protein (dStability > 0), so the residual activity of both D154K and the WT after exposure to different temperatures and incubation times was measured. The results of thermal stability analysis showed that both D154K and the WT exhibited comparable stability at temperatures ranging from 30 °C to 40 °C. Specifically, incubation at 30 °C for 1 h made no significant difference to enzyme activity for either protein. However, when incubated at 40 °C, both proteins showed a notable decrease in activity of about 40% (Figure 7a). Above 50 °C, the stability of D154K fell sharper than the WT, with it being completely inactivated at 60 °C. When the proteins were incubated at 37 °C for 2 to 12 h, the residual activity of D154K remained largely consistent with that of the WT, with both maintaining over 60% activity (Figure 7b). These findings suggested that while the D154K mutant exhibited slightly reduced thermal stability at higher temperatures, its stability under typical physiological conditions (30 °C to 40 °C) was comparable with that of the wild-type enzyme.

These experimental results indicated that the D154K mutation did not significantly affect the overall stability of the enzyme under typical physiological temperatures, suggesting that the mutant remained stable enough for practical applications in enzyme catalysis at physiological temperatures.

### 3.6. Effect of pH on rpHMGR Activity

**Figure 8:**
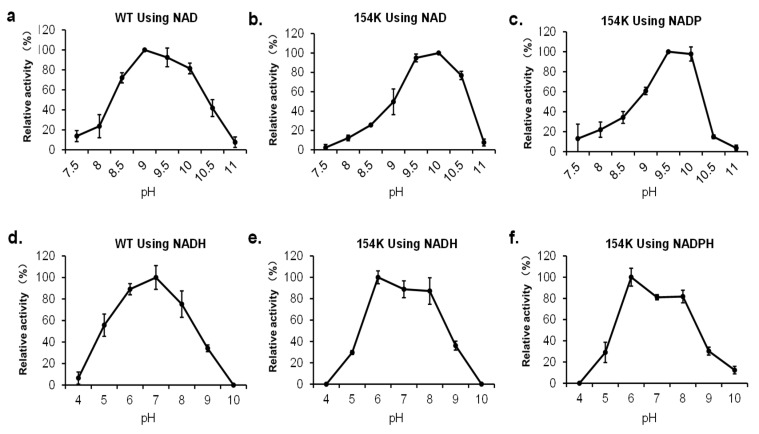
Effect of pH on the activity of *rp*HMGR: (**a**) activity of wild-type (WT) in the reverse reaction (oxidation of mevalonate) toward NAD; (**b**) activity of D154K in the reverse reaction toward NAD; (**c**) activity of D154K in the reverse reaction toward NADP; (**d**) activity of wild-type (WT) in the forward reaction (reduction of HMG-CoA) toward NADH; (**e**) activity of D154K in the forward reaction toward NADH; (**f**) activity of D154K in the forward reaction toward NADPH. Experiments were performed in triplicate, and error bars indicate the standard deviation.

Both forward and reverse reactions using the WT and D154K were performed to verify the influence of pH under varying conditions (Figure 8). A reductive deacylation of HMG-CoA, the forward reaction, was more likely to occur under neutral conditions (pH 6–8), while the oxidative acylation of mevalonate, the reverse reaction, preferred to take place under conditions that were more alkali (pH 9–10). The introduced substitution did not alter this property, but slight differences were observed. Referring to the oxidation of mevalonate, the WT exhibited a high activity period varying from pH8.5 to 10 of over 70%. For D154K, that period narrowed to 9.5 to 10.5 for NAD and 9.5 to 10 for NADP, and its activity dropped sharply beyond the edge (Figure 8a–c). For the reduction of HMG-CoA, both the WT and D154K showed high relative activity over 75% from pH6 to 8, while the most suitable pH changed from 7 to 6 after the mutation, whatever the cofactor was. Yet, D154K still maintained over 80% and 85% relative activity using NADPH and NADH at pH7, respectively (Figure 8d–f). In summary, D154K acted almost the same as the WT under varying pH conditions and the substitution did not weaken its activity. These results indicated that D154K could be a promising biocatalyst for the reductive deacylation of HMG-CoA under physiological conditions.

## 4. Conclusions

Terpenoids, with their diverse biological activities, have emerged as key targets in synthetic biology for biosynthetic pathway engineering and drug discovery. HMGR is a pivotal protein in the biosynthesis pathway of terpenes. To enhance the production of terpenoids through MVA pathways, engineering the cofactor flexibility of HMGR is a promising strategy. This study successfully explored the heterologous expression and characterization of *rp*HMGR from *R. pomeroyi* in *E. coli* and gained an intact enzyme by optimizing the expression conditions. The homology model of *rp*HMGR provided insights into its conserved amino acids and structural domains, combined with sequence alignment and MOE-assisted design, leading to the identification of D154K, a mutant that enhanced cofactor promiscuity. It exhibited a 53.7-fold increase in enzymatic activity with NADPH while maintaining substantial activity with NADH, demonstrating that this residue played a critical role in cofactor promiscuity. The mutant enzyme exhibited good stability and activity under physiological conditions of both temperature and pH. These findings highlight the potential of D154K for terpenoid biosynthesis in microbial cell factories. This study enhances the understanding of HMGR-cofactor recognition mechanisms and provides a foundation for future efforts to optimize and engineer HMGR for broader cofactor flexibility. Furthermore, the rational design approach demonstrated here could also offer valuable insights to engineer other oxidoreductases with expanded cofactor utilization capacity.

## Data Availability

Data are contained within the article and Supplementary Materials.

## Supplementary Materials

The following supporting information can be downloaded at https://www.mdpi.com/article/10.3390/biom15070976/s1, Table S1: Strains and plasmids used in this study; Table S2: Primers used in this study; Table S3: rpHMGR DNA sequences; Table S4: Virtual screening to compute dAffinity and dStability at 154 site; Figure S1: Original unprocessed Western blot membrane for rpHMGR crude enzyme solution; Figure S2: Homology modeling of rpHMGR using pmHMGR (PDB ID: 4I4B) as a template; Figure S3: Specific activities of wild-type (WT) and D154K in the reverse reaction.

## Abbreviations

The following abbreviations are used in this manuscript:

HMGR	3-Hydroxy-3-methylglutaryl-CoA Reductase
NAD	Nicotinamide Adenine Dinucleotide
NADP	Nicotinamide Adenine Dinucleotide Phosphate
