# Supplementary material for: Improving Cofactor Promiscuity of HMG-CoA Reductase from Ruegeria pomeroyi Through Rational Design
Source: Biomolecules. 2025 Jul 7;15(7):976. doi: 10.3390/biom15070976 (PMC12292504; doi:10.3390/biom15070976)
Supplement: Supplementary file 1 [file biomolecules-15-00976-s001.zip › biomolecules-3647763-supplementary.pdf]

Supplementary Material

Improving Cofactor Promiscuity of HMG-CoA Reductase from *Ruegeria pomeroyi* through Rational Design

Contents

Table S1. Strains used in this study

Table S2. Primers used in this study.

Table S3. rpHMGR DNA sequences

Table S4. Virtual screening to compute dAffinity and dStability at 154 site

Figure S1. Original unprocessed Western blot membrane for rpHMGR crude enzyme solution.

Figure S2. Homology modeling of *rp*HMGR using *pm*HMGR (PDB ID: 4I4B) as a template

Figure S3. Specific activities of wild-type (WT) and D154K in the reverse reaction

Table S1 Strains and plasmids used in this study

Strains and plasmids	Genotype or characteristic	Resource
Strains		
Escherichia coli BL21(DE3)	F-, *dcm, ompT, hsdS (rB-, mB-), gal,* *λ*(DE3)	Novagen
Plasmids		
pET28a(+)	Empty protein expression vector，Kan^R	Novagen
pET28a-rpHMGR(N)	pET28a(+) with rpHMGR wildtype, N-terminal 6×His tag, Kan^R	This study
pET28a-rpHMGR(C)	pET28a(+) with rpHMGR wildtype, C-terminal 6×His tag, Kan^R	This study
pET28a-rpHMGR-D154K	pET28a(+) with rpHMGR mutant D154K, N-terminal 6×His tag, Kan^R	This study

Table S2. Primers used in this study.

Primers	Sequence (5' to 3')
(-pET)rpHMGR-N-his-F1	GTTTAACTTTAAGAAGGAGATATACCATGCATCATCATCATCATCATACCGGCAAAACCGGTCATATTG
(-pET)rpHMGR-N-HISR	GCTTCCTTTCGGGCTTTGTTAGCAGCCGGATCCTAGGTATTTTCCAGAACCTGTTTTGC
rpHMGR-D154KF	CATGGAAGCAGCCGATGCAGTGAAACCGGTGCTGGTTGGTCTGGGC
rpHMGR-D154KR	GCCCAGACCAACCAGCACCGGTTTCACTGCATCGGCTGCTTCCATG
rpHMGR-D154F	CATGGAAGCAGCCGATGCAGTGNNKCCGGTGCTGGTTGGTCTGGGC
rpHMGR-D154R	GCCCAGACCAACCAGCACCGGMNNCACTGCATCGGCTGCTTCCATG

Table S3. rpHMGR DNA sequences

name	Sequence (5’ to 3’)
*rp*HMGR	ATGACCGGCAAAACCGGTCATATTGATGGCCTGAATAGTCGTATTGAAA<br>AAATGCGTGATCTGGATCCGGCCCAGCGTCTGGTGCGTGTGGCCGAAG<br>CCGCAGGTCTGGAACCGGAAGCAATTAGCGCCCTGGCAGGCAATGGCG<br>CCCTGCCTCTGAGCCTGGCCAATGGTATGATTGAAAATGTGATTGGCAA<br>ATTTGAGCTGCCGCTGGGCGTGGCCACCAATTTTACCGTTAATGGTCGC<br>GATTATCTGATTCCGATGGCCGTTGAAGAACCGAGCGTTGTGGCCGCCG<br>CCAGCTATATGGCACGCATTGCCCGTGAAAATGGTGGTTTTACCGCCCA<br>CGGTACCGCACCGCTGATGCGCGCTCAGATTCAGGTGGTTGGTCTGGG<br>TGACCCGGAAGGTGCCCGCCAGCGTCTGCTGGCACATAAAGCCGCATT<br>CATGGAAGCAGCCGATGCAGTGGATCCGGTGCTGGTTGGTCTGGGCGG<br>CGGTTGCCGTGATATTGAAGTTCATGTGTTTCGTGATACCCCGGTTGGC<br>GCAATGGTTGTTCTGCATCTGATTGTGGATGTGCGTGATGCAATGGGTG<br>CAAATACCGTGAATACAATGGCTGAACGCCTGGCACCGGAAGTTGAAC<br>GTATTGCAGGTGGCACCGTGCGCCTGCGCATTCTGAGCAATCTGGCAG<br>ATCTGCGCCTGGTTCGCGCACGCGTGGAACTGGCCCCGGAAACCCTGA<br>CCACCCAGGGTTATGATGGCGCCGATGTGGCACGCGGTATGGTTGAAG<br>CATGCGCCCTGGCAATTGTGGATCCGTATCGTGCAGCCACCCATAATAA<br>GGGCATTATGAATGGCATTGATCCGGTGGTTGTTGCCACCGGTAATGATT<br>GGCGCGCCATTGAAGCAGGCGCCCATGCATATGCAGCACGCACCGGCC<br>ATTATACCAGCCTGACCCGTTGGGAACTGGCAAATGATGGTCGCCTGGT<br>GGGTACCATTGAACTGCCGCTGGCACTGGGTCTGGTTGGTGGCGCAAC<br>CAAAACCCATCCGACCGCACGCGCCGCCCTGGCATTAATGCAGGTTGA<br>AACCGCAACCGAACTGGCACAGGTGACCGCAGCCGTTGGTCTGGCAC<br>AGAATATGGCCGCAATTCGCGCACTGGCCACCGAAGGTATTCAGCGTG<br>GTCATATGACCCTGCATGCACGCAATATTGCCATTATGGCCGGTGCCACC<br>GGCGCAGATATTGATCGCGTGACCCGTGTTATTGTTGAAGCAGGCGATG<br>TTAGTGTTGCACGCGCAAAACAGGTTCTGGAAAATACC

Table S4. Virtual screening to compute dAffinity and dStability at 154 site

mutation	dAffinity (kcal/mol)	dStability(kcal/mol)
D154D	0	0
D154A	3.6918	1.1064
D154R	-61.5323	1.1152
D154N	6.0467	1.4267
D154C	37.9306	1.1398
D154Q	-23.6233	0.6298
D154E	1.1836	-0.191
D154G	-35.2087	1.9473
D154H	36.3949	0.4517
D154I	-7.8864	-0.6060
D154L	-50.3338	-0.5630
D154K	-73.4067	1.6791
D154M	-62.6761	0.0108
D154F	100.1022	0.1182
D154P	-200.08	1.0556
D154S	58.8865	1.1636
D154T	-0.5635	0.5363
D154W	195.3395	0.6979
D154Y	70.2977	0.6453
D154V	-60.0638	0.2188

Note: dAffinity<0 denotes an increase in affinity compared to the wild-type, and dStability<0 denotes an increase in stability compared to the wild-type

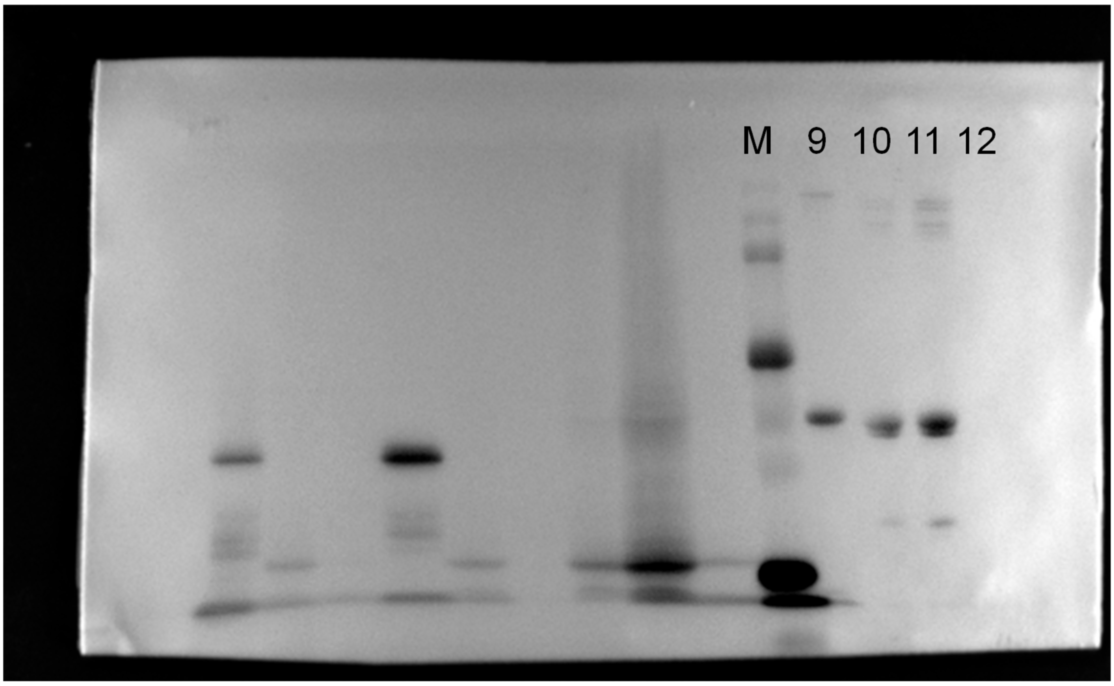

Figure S1. Original unprocessed Western blot membrane for rpHMGR crude enzyme solution. Lane 9: rpHMGR-C; lanes 10 and 11: rpHMGR-N; lane 12: negative control with strain carrying empty pET28a(+) vector.

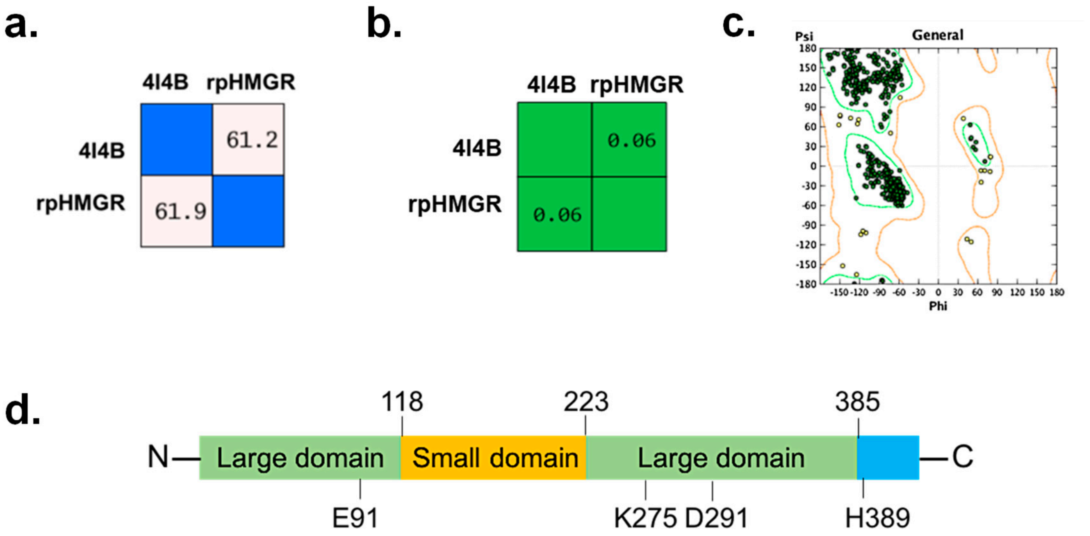

Figure S2. Homology modeling of *rp*HMGR using *pm*HMGR (PDB ID: 4I4B) as a template (a) Sequence identity of *pm*HMGR and *rp*HMGR; (b) RMSD of *pm*HMGR and *rp*HMGR (c)Ramachandran plot of *rp*HMGR using *pm*HMGR as a template homology modeling; (d) Representation of different structural domains and conserved amino acid residues in the primary structure of *rp*HMGR: green for the large domain, orange for the small domain, and blue for the C-terminal flap domain.

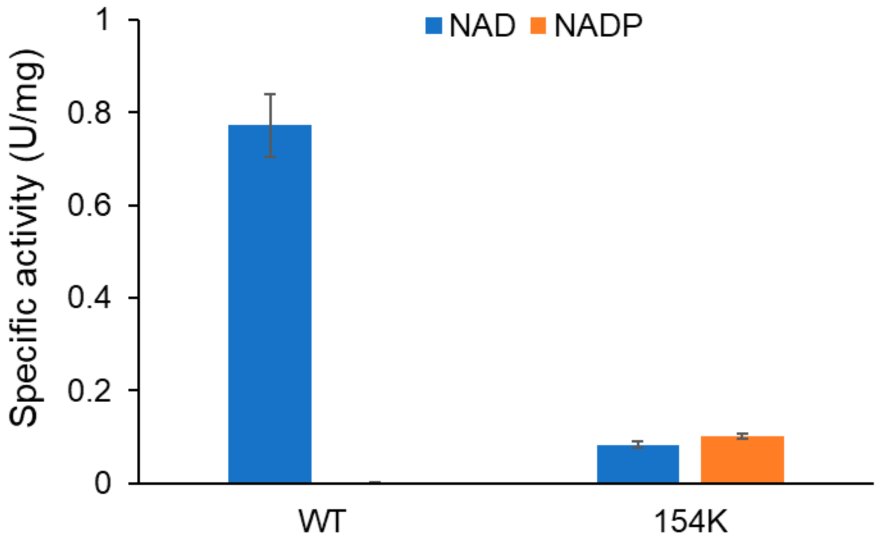

Figure S3. Specific activities of wild-type (WT) and D154K in the reverse reaction (oxidative acylation of mevalonate) towards NAD and NADP, experiments were done in triplicate, and error bars indicate standard deviation.
